# 6 Minute Walk Test in Duchenne MD Patients with Different Mutations: 12 Month Changes

**DOI:** 10.1371/journal.pone.0083400

**Published:** 2014-01-08

**Authors:** Marika Pane, Elena S. Mazzone, Maria Pia Sormani, Sonia Messina, Gian Luca Vita, Lavinia Fanelli, Angela Berardinelli, Yvan Torrente, Adele D'Amico, Valentina Lanzillotta, Emanuela Viggiano, Paola D'Ambrosio, Filippo Cavallaro, Silvia Frosini, Luca Bello, Serena Bonfiglio, Roberta Scalise, Roberto De Sanctis, Enrica Rolle, Flaviana Bianco, Marlene Van der Haawue, Francesca Magri, Concetta Palermo, Francesca Rossi, Maria Alice Donati, Chiara Alfonsi, Michele Sacchini, Maria Teresa Arnoldi, Giovanni Baranello, Tiziana Mongini, Antonella Pini, Roberta Battini, Elena Pegoraro, Stefano C. Previtali, Sara Napolitano, Claudio Bruno, Luisa Politano, Giacomo P. Comi, Enrico Bertini, Lucia Morandi, Francesca Gualandi, Alessandra Ferlini, Nathalie Goemans, Eugenio Mercuri

**Affiliations:** 1 Department of Paediatric Neurology, Catholic University, Rome, Italy; 2 Biostatistics Unit, Department of Health Sciences, University of Genoa, Italy; 3 Department of Neurosciences, Psychiatry and Anaesthesiology, University of Messina, Messina, Italy; 4 Child Neurology and Psychiatry Unit, “C. Mondino” Foundation, Pavia, Italy; 5 Dino Ferrari Centre, Neuroscience Section, Department of Pathophysiology and Transplantation (DEPT), University of Milan, Neurology Unit, Ca' Granda Ospedale Maggiore Policlinico, Milan, Italy; 6 Unit of Neuromuscular and Neurodegenerative Diseases, Department of Neurosciences, Bambino Gesù Children's Hospital, Rome, Italy; 7 Neuromuscular Disease Unit, G. Gaslini Institute, Genoa, Italy; 8 Dipartimento di Medicina Sperimentale, Seconda Università di Napoli, Napoli, Italy; 9 Department of Developmental Neuroscience, Stella Maris Institute, University of Pisa, Pisa, Italy; 10 Department of Neurosciences, University of Padua, Padua, Italy; 11 Child Neurology and Psychiatry Unit, IRCCS Istituto delle Scienze Neurologiche, Bologna, Italy; 12 Neuromuscular Center, SG. Battista Hospital, University of Turin, Turin, Italy; 13 Pediatric Neurology KU Gasthuisberg, Leuven, Belgium; 14 Metabolic and Neuromuscular Unit, Meyer Hospital, Florence, Italy; 15 Developmental Neurology Unit, Istituto Neurologico “Besta”, Milan, Italy; 16 Department of Neurology, San Raffaele Scientific Institute, Milan, Italy; 17 Neuromuscular Disease and Immunology Unit, Istituto Neurologico “Besta”, Milan, Italy; 18 Section of Medical Genetics, Department of Experimental and Diagnostic Medicine, University of Ferrara, Ferrara, Italy; The Hospital for Sick Children, Canada

## Abstract

**Objective:**

In the last few years some of the therapeutical approaches for Duchenne muscular dystrophy (DMD) are specifically targeting distinct groups of mutations, such as deletions eligible for skipping of individual exons. The aim of this observational study was to establish whether patients with distinct groups of mutations have different profiles of changes on the 6 minute walk test (6MWT) over a 12 month period.

**Methods:**

The 6MWT was performed in 191 ambulant DMD boys at baseline and 12 months later. The results were analysed using a test for heterogeneity in order to establish possible differences among different types of mutations (deletions, duplications, point mutations) and among subgroups of deletions eligible to skip individual exons.

**Results:**

At baseline the 6MWD ranged between 180 and 560,80 metres (mean 378,06, SD 74,13). The 12 month changes ranged between −325 and 175 (mean −10.8 meters, SD 69.2). Although boys with duplications had better results than those with the other types of mutations, the difference was not significant.

Similarly, boys eligible for skipping of the exon 44 had better baseline results and less drastic changes than those eligible for skipping exon 45 or 53, but the difference was not significant.

**Conclusions:**

even if there are some differences among subgroups, the mean 12 month changes in each subgroup were all within a narrow Range: from the mean of the whole DMD cohort. This information will be of help at the time of designing clinical trials with small numbers of eligible patients.

## Introduction

In the last few years several therapeutical approaches have become available for patients affected by Duchenne muscular dystrophy (DMD) [1]–[4]. Some of these approaches are specifically targeting distinct types of mutations, such as nonsense mutations, causing premature stop codons, or specific deletions as in exon skipping studies [5]–[9]. Because of this, the number of patients eligible for each study is limited to those having the targeted mutations and is further restricted by other inclusion criteria such as age or functional level. In the first studies using antisense oligonucleotides, the first exon to be targeted was exon 51 as this had the highest number of patients eligible for its skipping [5], [8], [10]–[12] (17% of the deletions reported in the Leiden database [13]). Ongoing or planned studies are targeting for skipping other exons (exons 44,53,45) with a lower number of potentially eligible DMD boys and increasing difficulties in recruiting sufficient numbers to allow placebo groups. This has raised the question whether natural history data could be used as controls and, more generally, whether individual groups of mutations have distinct profiles of progression that may be different from what observed in the whole DMD cohort.

Longitudinal natural history data collected in relatively large cohorts of DMD ambulant boys [14]–[16] are available but no systematic study has been performed to explore functional changes in individual groups of mutations.

The aim of this study was to report 12 month changes of 6MWT in a large cohort of DMD ambulant patients subdivided according to groups of mutations.

## Subjects and Methods

The patients reported are part of longitudinal multicentric cohort studies involving 13 tertiary neuromuscular centers in Italy and one centre in Belgium. Patients were recruited from January 2008 and followed for at least one year. The study was approved by the Ethical Committees of the Catholic University, Rome, Ospedale Bambino Gesù, Rome, Istituto Mondino, Pavia, Gaslini Institute, Genova, Besta Institute, Milan, Stella Maris Institute, Pisa, Ospedale Maggiore, Bologna, University of Napoli, University of Messina, University of Torino, University of Padova, University of Milano, University of Leuven. As the assessment was already part of the clinical routine in all centers, with the approval of the Ethics Committees, verbal consent to record the anonymized data in a database was obtained by the parents for the boys under age.

Patient inclusion criteria at baseline were: genetically proven DMD diagnosis, patient still ambulant and able to walk independently for at least 100 meters, no severe or moderate learning difficulties or behavioral problems. Genetic and treatment information were collected and classified following the criteria used in our previous studies on the same cohort [15], [16]. The study was approved by the ethical committee of each center.

We broadly subdivided our cohort into patients on steroids and those with no steroid treatment, this included boys who had never been on steroids and others who had used them for less than a year and had stopped treatment at least one year before the study.

### 6MWT

6MWT was performed in all DMD ambulant boys according to the ATS guidelines [17], modified by having two examiners, one recording time and distances and one staying close to the patient for safety issues. As part of the routine assessments in all centers patients are seen at least once every 12 months. Data were collected from the first assessment after recruitment (baseline) and from the 12 month assessment. Details of the training and interobserver reliability have been previously reported [15], [16].

### Statistical analysis

The 6MWT was evaluated longitudinally over a 12-month period of time. Summary statistics (N, mean, SD, Range:) were used. Both baseline 6MWT and its 12 months change were symmetrically distributed around their mean value and the distribution is close to a normal one.

Furthermore, the 6 minute walk distance (6MWD) data were converted to a percent predicted (%-predicted) value according to the age and height based Geiger equation (% Geiger = 196.72+39.81*age−(1.36*age^2^)+132.28 *height(m)) as has been proposed to account for growth and maturational influences [18], [19] The same descriptive statistics were calculated as for the 6MWD.

Both 6MWD and % predicted data were subdivided according to the type of deletion (deletions, duplications, point mutations). Boys with deletions were further subdivided identifying those carrying mutations eligible for skipping of the exons that, according to the Leiden database, are more frequent in the DMD population (see Table S1).

An ANOVA model adjusting for baseline age was used to assess heterogeneity among groups of 6MWD baseline values and its 12 months change. The test was performed on the whole cohort and in the group who had deletions. As the recent clinical trials only include patients who are on steroids we also performed the test for heterogeneity excluding the boys who were not on steroids.

## Results

One hundred and ninety-one patients fulfilled the inclusion criteria and entered the study. All had out of frame mutations and/or absent dystrophin on muscle biopsy.

113 of these patients have already been reported in our previous studies reporting follow up data in ambulant DMD boys [15]. Of the 191 boys, 176 were on steroids, and 15 were not on steroids.

132 had deletions, 15 had duplications and 44 point mutations. The distribution of groups of deletions according to their eligibility to exon skipping are shown in table 1 and in the online additional table.

**Table:** Table 1. Baseline and 12 month changes of 6MWD in the whole DMD cohort and in different mutation subgroups.

	Age	6MWD Baseline	6MWD 12 month changes	% predicted Baseline	% predicted 12 month changes
Whole cohort (n = 191)	Range: 3,2 to 15,0 Mean 7,90 SD 2,23	Range: 185 to 560,80 Mean 378,06 SD 74,13	Range: −325 to 175 Mean −10,19 SD 69,33	Range: 0,28 to 1,19 Mean 0,66 SD 0,15	Range: −71 to 39 Mean −3,88 SD 12,57
Below age 7 (n = 80)	Range: 3,2 to 7 Mean 5,84; SD 0,9	Range: 215 to 521,50 Mean 383,09 SD 64,4	Range: −95 to 175 Mean 27,37 SD 53,02	Range: 0,39 to 1,19 Mean 0,73 SD 0,13	Range: −71 to 39 Mean 1,09 SD 13,81
Above age 7 (n = 111)	Range: 7,1 to 15,0 Mean 9,38 SD 1,7	Range: 185 to 560,80 Mean 374,43 SD 80,5	Range: −325 to 102 Mean −37,25 SD 67,21	Range: 0,28 to 0,96 Mean 0,62 SD 0,14	Range: −50 to 16 Mean −7,36 SD 10,25
Duplications (n = 15)	Range: 5,9 to 15 Mean 8,6 SD 2,28	Range: 187 to 521 Mean 420,26 SD 85,3	Range: −87 to 102 Mean 4,37 SD 54,14	Range: 0,29 to 0,97 Mean 0,72 SD 0,17	Range: −15 to 14 Mean −1,53 SD 8,40
Point mutations (n = 44)	Range: 4,4 to 12,6 Mean 7,5 SD 1,9	Range: 250 to 560,80 Mean 393,62 SD 70,53	Range: −135 to 164 Mean −0,84 SD 53,60	Range: 0,40 to 1,19 Mean 0,70 SD 0,16	Range: −24 to 26 Mean −2,65 SD 8,67
All deletions (n = 132)	Range: 3,2 to 13,7 Mean 7,96 SD 2,32	Range: 185 to 557,50 Mean 368,07 SD 71,93	Range: −325 to 175 Mean −14,95 SD 75,09	Range: 0,28 to 0,95 Mean 0,65 SD 0,14	Range: −71 to 39 Mean −4,41 SD 13,83
*Deletions eligible for skipping exon 44 (n = 18)	Range: 4,6 to 13 Mean 8,2 SD 2,6	Range: 300 to 557,50 Mean 398,16 SD 65,28	Range: −84 to 118 Mean −11,78 SD 54,89	Range: 0,51 to 0,95 Mean 0,68 SD 0,11	Range: −16 to 15 Mean −4 SD 8,22
Deletions eligible for skipping exon 45 (n = 15)	Range: 5 to 11,4 Mean 8,4 SD 2,35	Range: 187 to 485 Mean 334,46 SD 72,46	Range: −325 to 135 Mean −21,6 SD 111,76	Range: 0,29 to 0,92 Mean 0,59 SD 0,16	Range: −71 to 21 Mean −10,16 SD 23,35
*Deletions eligible for skipping exon 46 (n = 7)	Range: 4,6 to 9,9 Mean 7,3 SD 2,37	Range: 185 to 419 Mean 335,30 SD 85,47	Range: −38 to 31 Mean 0,83 SD 24,51	Range: 0,28 to 0,85 Mean 0,59 SD 0,19	Range: −7 to 1 Mean −1,83 SD 2,93
Deletions eligible for skipping exon 50 (n = 9)	Range: 4,1 to 10,8 Mean 7,4 SD 2,33	Range: 256 to 477,50 Mean 358,63 SD 77,11	Range: −55 to 83 Mean −7,56 SD 44,84	Range: 0,49 to 0,79 Mean 0,65 SD 0,11	Range: −10 to 11 Mean −3,63 SD 6,97
**Deletions eligible for skipping exon 51 (n = 27)	Range: 3,6 to 12,5 Mean 7,7 SD 2,17	Range: 200 to 462 Mean 362,66 SD 62,26	Range: −246 to 99 Mean −21,59 SD 76,33	Range: 0,31 to 0,79 Mean 0,63 SD 0,12	Range: −40 to 13 Mean −5,19 SD 11,62
**Deletions eligible for skipping exon 53 (n = 28)	Range: 5 to 13 Mean 8,6 SD 1,96	Range: 189 to 458 Mean 344,11 SD 67,16	Range: −240 to 127 Mean −34,18 SD 77,99	Range: 0,3 to 0,83 Mean 0,60 SD 0,13	Range: −37 to 20 Mean −6,89 SD 12,10

4 boys had deletion of exon 45 and were included both in the group eligible for skipping 44 and 46.

x boys had deletion of exon 52 and were included both in the group eligible for skipping 51 and 53.

### 6MWT

Four children lost the ability to perform the test within 12 months. At baseline the 6MWD ranged between 180 and 560,80 metres (mean 378,06, SD 74,13). Converting the 6MWD to % predicted values, based on the Geiger equation, the baseline values ranged between 28.31 and 119.49 with an average of 66.5 (SD 14.71).

The 12 month changes ranged between −325 and 175 with an average of −10.8 meters (SD 69.2). The 12 month % predicted ranged between −71 and 39 with an average of −4 (SD 12.76) (table 1)

### Type of mutations

Table 1 show details of the 6MWD at baseline and of the 12 months changes. There was no significant difference between deletions, duplications and point mutations neither at baseline (Figure S1a) nor in the 12 month changes (Figure S1b)

### Groups of deletions and 6MWT

**Figure 1:**
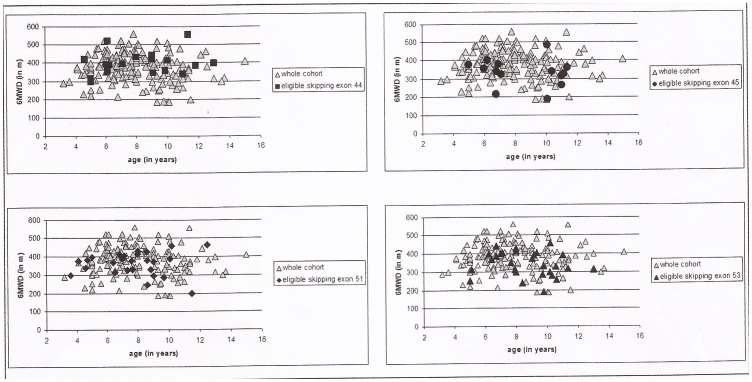
Baseline values of 6MWD: in each graph the individual results obtained in individual subgroups (eligible for skipping exon 44, 45, 51 and 53) are plotted against those found in the whole DMD color.

**Figure 2:**
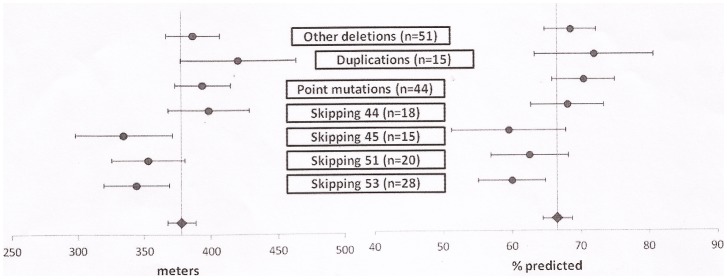
Mean raw scores (left panel) and % predicted (right panel) of 6MWD in individual subgroups.

Figure 1 shows details of the 6MWD at baseline in the different subgroups of deletions identified according to their eligibility to skip specific exons. A statistically significant heterogeneity was detected among groups with a deletion (p = 0.03) (figure 2). The heterogeneity was no longer significant when baseline values were converted to % predicted (p = 0.06).

**Figure 3:**
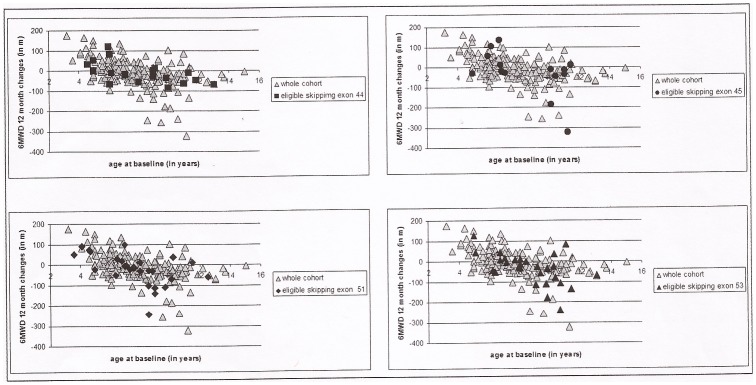
Twelve month changes of 6MWD: in each graph the individual results obtained in individual subgroups (eligible for skipping exon 44, 45, 51 and 53) are plotted against those found in the whole DMD color.

Figure 3 shows details of the 12 month changes in the 6MWD in the different subgroups of deletions identified according to their eligibility to skip specific exons.

**Figure 4:**
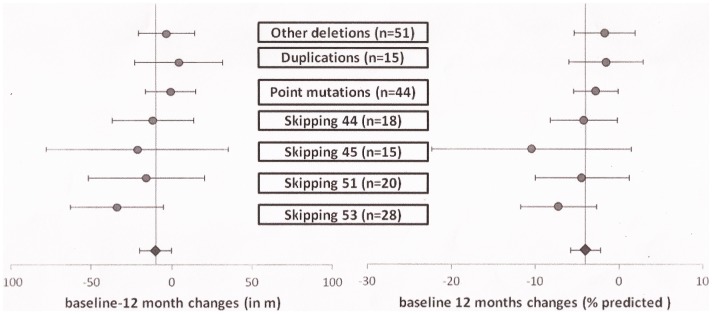
Mean 12 month changes (left panel) and % predicted (right panel) of 6MWD in individual subgroups.

On the test for heterogeneity there were no differences among the groups (p = 0.53) also adjusting for age (p = 0.70). The heterogeneity among the groups is not significant also when only the groups with a deletion were considered (p = 0.86 for heterogeneity adjusting for age) (figure 4).

This was true also when the raw scores were converted in % predicted (p = 0.33 for all the groups and p = 0.47 within the deletion group).

The results of the test after excluding the boys who were not on steroids showed similar results to the whole cohort.

## Discussion

Recent natural history longitudinal studies using the 6MWT and the NSAA to measures changes in ambulant DMD boys have reported a wide variability of such changes over 12 and 24 months [15], [16]. The recent advent of therapeutical approaches targeting specific groups of mutations has highlighted the need to establish whether some of the variability observed may be due to the different mutations in the dystrophin gene.

In our cohort boys with duplications and point mutations (single nucleotide substitutions) had better overall scores than those with deletions at baseline but the difference was not significant. The 12 month changes showed even less difference among the three types of mutations than at baseline. These findings are in agreement with previous studies, also reporting that the type of mutations does not influence clinical evolution [20]. In our cohort the incidence of point mutations was higher than that reported in the Leiden database [13] and reflect a general trend also observed in our Italian registry, that also includes younger and non ambulant boys not included in this study (data not shown).

In this paper we were specifically interested to the groups of deletions identified according to their eligibility to skip individual exons. In agreement with the Leiden database [13], we also found that the subgroups of mutations eligible for skipping exons 51, 53, 44 and 45 were the most frequent [13]. In our cohort however, those eligible for skipping exon 53 were relatively more frequent and those eligible for skipping of exon 45 less frequent than in the Leiden database.

The results of our study highlighted two important findings: there are some differences among individual subgroups but, on the test for heterogeneity this difference was not significant, neither at baseline nor on the 12 month changes.

More specifically the subgroup eligible for skipping exon 44 had overall better baseline values of 6MWD than the other subgroups, and the difference was also observed when the raw scores were converted to % predicted. On the 12-month changes the difference was smaller. The subgroup eligible for skipping exon 45 and 53 in contrast appeared to have overall lower baseline values and more negative 12 month changes than the other subgroups. These results should be interpreted with caution because of the relatively small numbers and the large standards deviations with a number of out layers who have probably influenced the results for each subgroup, and because there are several reasons that may account for such differences. The use of the % predicted helped to reduce the risk that age or height could have influenced the analysis in relatively small groups because of growth. We did not specifically addressed the issue of steroids as this was addressed in our recent paper on the same cohort, but, with few exceptions, all the boys in the present study were on steroids and with a similar distribution of daily and intermittent regimes in each subgroup. When we excluded from the analysis the boys who were not on steroids, the level of significance did not change.

It is not completely surprising that the deletions eligible for skipping of the exon 44 had better results than other subgroups as there is reported evidence that out-of-frame deletions around exons 44 can have a high rate of “exceptions” to the reading frame rule resulting in a quite wide range of clinical severity, including intermediate (IMD) or even BMD and therefore better baseline values on the 6MWT [21], [22]. Furthermore it has also been recently suggested that DMD patients with deletions flanking exon 44 have significantly higher percentage of revertant fibres and traces of low-level dystrophin expression than those with deletions surrounding exon 51 [23], and this could also explain the relatively better baseline values.

Further help in clarifying the variability of findings may also come from the analysis of specific genotypes, such as the SPP1or the LTBP4 genotype, that have recently been found to contribute to predict the severity of the phenotype in DMD [24] [25].

This study provides for the first time information on the natural history of distinct groups of mutations using the same outcome measure (6MWT) and the same time frame (12 months), that have been chosen in the recent and ongoing clinical trials in ambulant DMD [12], [26]. Another advantage of this study is that we included a larger proportion of boys below the age of 7 years than in the other recent natural history studies, therefore providing further information of the 6MWD in the younger children who, as also suggested by the regulatory authorities, may be a good target for efficacy studies as their muscles are more preserved. The larger number of younger children is also reflected in the results, showing less negative 12 month 6MWD changes than those previously obtained in cohorts with a smaller proportion of DMD boys younger than 7 years [15], [16].

In conclusion our results showed some differences among subgroups, that were more obvious at baseline, with patients eligible for skipping 44 having overall better results than the others. The difference between individual subgroups (expecially 44 versus 45 or 53) however was bigger than the difference between each subgroup and the whole cohort of DMD boys. The 12 month mean changes in each subgroup were all within a relatively narrow range from the mean of the whole DMD cohort as also proved by the test for heterogeneity that did not show a significant difference. Rather than focusing on the non-significance of the heterogeneity test among subgroups that is favoured by the relatively small numbers of subjects included in each subgroup, the important issue is that this paper for the first time provides an estimate of the size of the variations that can be expected in subgroups due to the different mutations in the dystrophin gene. This information will be of help at the time of designing clinical trials with small numbers of eligible patients.

This paper also suggest that age, height and steroids cannot fully explain the variability of the findings and further studies on larger cohorts, exploring modifying genes and correlating functional abilities with dystrophin analysis are needed to further clarify the variability observed in the whole cohort and identify criteria for stratification and possible adjustments.

## Supporting Information

Figure S1 **Baseline values (left panel) and 12 month changes of 6MWD in different types of mutations.**
(TIF)

Table S1 **Online additional table (S1): Table reporting the mutations eligible for skipping of individual exons in the Leiden database and in the present study.**
(DOC)
