# Supplementary figures and images for: 6 Minute Walk Test in Duchenne MD Patients with Different Mutations: 12 Month Changes
Source: PLoS One. 2014 Jan 8;9(1):e83400. doi: 10.1371/journal.pone.0083400 (PMC3885414; doi:10.1371/journal.pone.0083400)

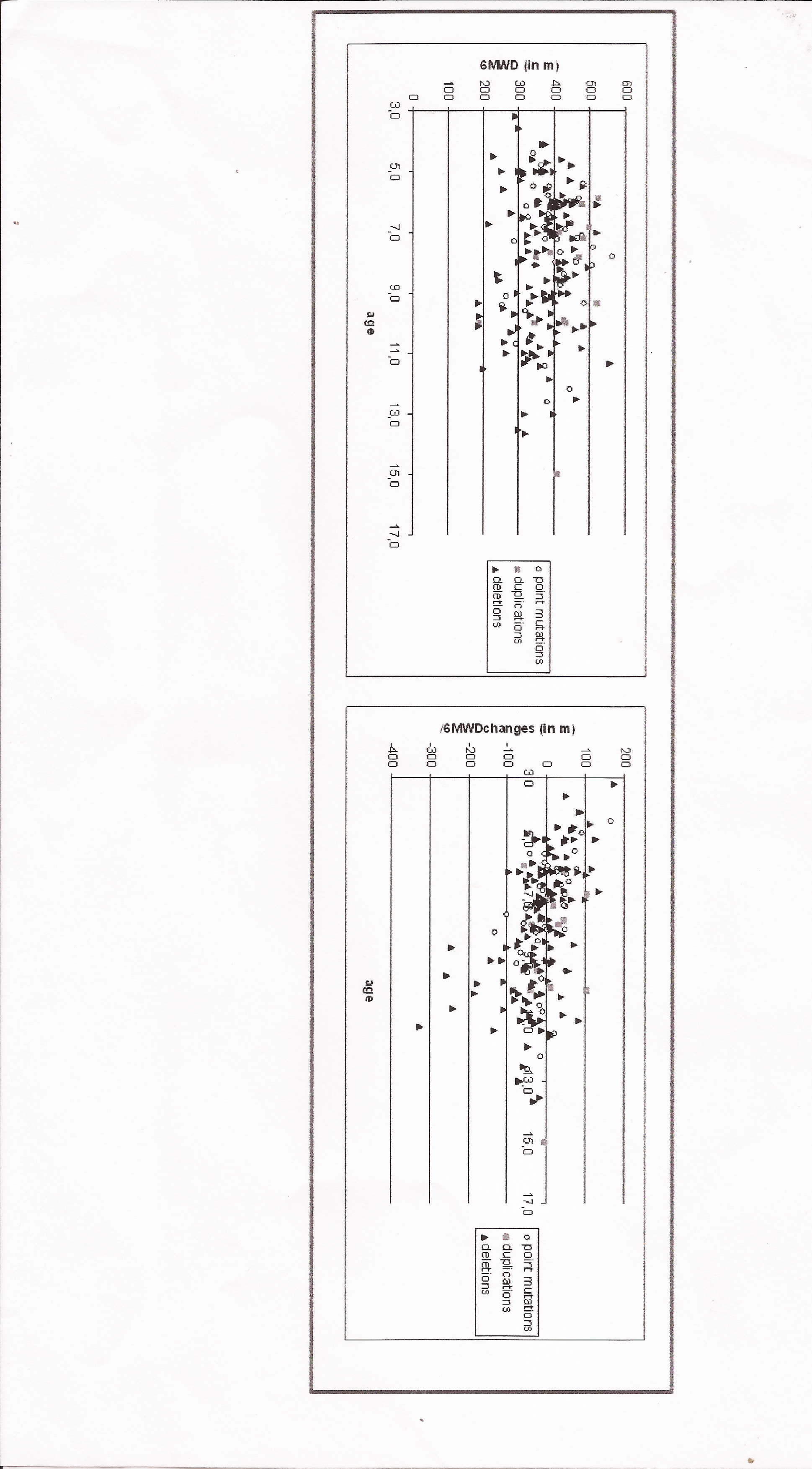

Supplement: Figure S1 — Baseline values (left panel) and 12 month changes of 6MWD in different types of mutations. (TIF) [file pone.0083400.s001.tif]
