# Supplementary material for: 6 Minute Walk Test in Duchenne MD Patients with Different Mutations: 12 Month Changes
Source: PLoS One. 2014 Jan 8;9(1):e83400. doi: 10.1371/journal.pone.0083400 (PMC3885414; doi:10.1371/journal.pone.0083400)
Supplement: Table S1 — Online additional table (S1): Table reporting the mutations eligible for skipping of individual exons in the Leiden database and in the present study. (DOC) [file pone.0083400.s002.doc]

Online additional table (S1)

| Skippable exon | Therapeutic for DMD deletions (exons) | % of deletions in the Leiden database | Present study |
| --- | --- | --- | --- |
| 2 | 3-7, 3-19, 3-21 | 2.9 | 1,04 |
| 8 | 3-7, 4-7, 5-7, 6-7 | 4.5 | 1,04 |
| 17 | 12-16, 18-33, 18-41, 18-44 | 1.8 | 0 |
| 43 | 44, 44-47, 44-49, 44-51 | 3.7 | 2,61 |
| 44 | 14-43, 19-43, 30-43, 35-43, 36-43, 40-43, 42-43, 45, 45-54 | 7.8 | 9,4 |
| 45 | 12-44, 18-44, 44, 46-47, 46-48, 46-49, 46-51,46-53, 46-55 | 11.2 | 7,85 |
| 46 | 21-45, 45, 47-54, 47-56 | 5.6 | 3,14 |
| 50 | 51, 51-53, 51-55 | 5.2 | 4,71 |
| 51 | 45-50, 47-50, 48-50, 49-50, 50, 52, 52-63 | 17.5 | 14,65 |
| 52 | 51-53, 53-55 | 4.0 | 1,04 |
| 53 | 10-52, 45-52, 47-52, 48-52, 49-52, 50-52, 52 | 7.5 | 14,73 |
| 55 | 45-54, 48-54 | 1.8 | 1,57 |

Table reporting the mutations eligible for skipping of individual exons in the Leiden database and in the present study
